# Elucidating the Potential Targets and Mechanisms of Bisphenol A-Induced Prostate Cancer Based on Network Toxicology and Molecular Docking Analyses

**DOI:** 10.32604/or.2026.076716

**Published:** 2026-04-22

**Authors:** Ashuai Du, Dianbin Guo, Dongbo Yuan, Kai Li, Yuanyuan Luo, Songsong Tan, Xuchao Dai, Bo Yu, Wanxiang You, Junjie Zhao, Bo Yan, Kehua Jiang, Xiaofei Fan, Jianguo Zhu

**Affiliations:** 1 Department of Infection, Guizhou Provincial People’s Hospital, Guiyang, China; 2 Shandong Medical College, No. 5460, Second Ring South Road, Jinan, China; 3 Department of Urology, Guizhou Provincial People’s Hospital, Guiyang, China; 4 Department of Urology, GuiZhou University Medical College, Guiyang, China; 5 Graduate School, Zunyi Medical University, Zunyi, China; 6 Department of Urology, Dejiang County People’s Hospital of Guizhou Province, Dejiang, China; 7 Yantai Yuhuangding Hospital, Yantai, China

**Keywords:** Bisphenol A, prostate cancer, network toxicology, phosphatidylinositol 3-kinase/protein kinase B (PI3K/AKT) signaling, LY294002

## Abstract

**Background:**

Bisphenol A (BPA) is a widely used industrial chemical and endocrine-disrupting compound, and accumulating evidence suggests that it may contribute to prostate cancer progression; however, the underlying molecular mechanisms remain incompletely elucidated. This study aimed to elucidate the molecular targets and signaling pathways underlying BPA-induced prostate cancer progression.

**Methods:**

In this study, an integrated strategy combining network toxicology, molecular docking, and molecular dynamics simulations was employed to identify potential BPA-related targets and signaling pathways involved in prostate cancer. Candidate targets were retrieved from public databases, followed by protein-protein interaction network analysis to screen key hub genes. Functional assays were performed to evaluate the effects of BPA on prostate cancer cell migration, invasion, epithelial-mesenchymal transition (EMT), and phosphatidylinositol 3-kinase/protein kinase B (PI3K/AKT) signaling, and an *in vivo* mouse model was used to assess the impact of BPA exposure and PI3K inhibition on tumor progression.

**Results:**

Eighteen BPA-related core targets were identified, among which androgen receptor (AR), matrix metalloproteinase 9 (MMP9), matrix metalloproteinase 2 (MMP2), kallikrein-related peptidase 3 (KLK3), and hypoxia-inducible factor 1 alpha (HIF1A) emerged as key hub genes. Computational analyses indicated stable predicted interactions between BPA and these proteins. Functionally, BPA exposure promoted prostate cancer cell invasion and EMT, which were associated with activation of the PI3K/AKT and MMP signaling pathways, whereas the PI3K inhibitor LY294002 effectively attenuated BPA-induced invasive phenotypes *in vitro* and reduced tumor progression *in vivo*.

**Conclusions:**

Collectively, these findings provide mechanistic insights into BPA-driven prostate cancer progression and highlight the value of network toxicology-based approaches in environmental toxicology research.

## 1 Introduction

Bisphenol A (BPA) is extensively used in food and beverage packaging, medical devices, thermal paper, and dental materials [1]. However, over the past 20 years, numerous animal and epidemiological studies have shown an association between BPA exposure and adverse health effects, including neurological and behavioral changes [2], obesity [3], reproductive disruption [4], mammary gland tumors [5], ovarian cancer [6], and colon cancer [7,8]. Moreover, recent studies have indicated that elevated BPA levels increase the risk of developing prostate cancer [9,10]. Consistent with its widespread use, BPA has been detected in human urine, serum, and blood at concentrations ranging from low nanomolar levels in the general population to higher levels in occupationally or environmentally exposed groups. Epidemiological and biomonitoring studies have linked BPA exposure to metabolic, reproductive, and cardiovascular disturbances [11–13]. Major exposure sources include food and beverage containers, epoxy resin can linings, thermal paper receipts, and certain medical or dental materials [14,15]. Despite regulatory restrictions, continuous low-level BPA exposure remains prevalent, highlighting the need to clarify its biological effects and molecular mechanisms in diseases such as prostate cancer.

Prostate cancer is the second most widely diagnosed cancer and the sixth leading cause of cancer-related death in males, with 248,530 new cases reported globally in 2021 [16]. Although the incidence of prostate cancer and associated mortality have stabilized following the introduction of prostate-specific antigen testing, risk factors such as age, ethnicity, family history, diet, and drug resistance remain significant [17–19]. Moreover, disease prevalence has been associated with environmental factors, including prolonged exposure to toxic bioaccumulating chemicals such as BPA [20]. However, advances in exposure assessments now offer more precise insights into the association between BPA and prostate cancer.

Network toxicology is an analytical framework that integrates network pharmacology, bioinformatics, and technologies such as genomics, proteomics, and metabolomics, to examine toxicological characteristics without the need for traditional animal-based toxicology experiments [21]. By using multiple databases to construct “Compound-Target-Disease” network models, this framework represents a simple and accurate approach that is broadly applicable in fields such as environmental health, food safety, pharmaceutical research, and disease prevention [22]. Molecular docking is a computational method that can be used to predict ligand-receptor binding interactions and associated affinities [23], facilitating the evaluation of interaction stability and likelihood, as lower binding energies are considered to contribute to more favorable interactions [24]. Such docking analyses are widely used in drug design and the assessment of compound-target interactions [25,26].

Collectively, these approaches can provide a robust theoretical framework for gaining an understanding of complex biological processes; thus, integrating network toxicology with molecular docking techniques offers a promising analytical strategy. Accordingly, we reasoned that a combination of these approaches would be useful for investigating the potential toxic mechanisms of BPA and key target proteins involved in prostate cancer. Therefore, the aim of this study was to systematically investigate the potential toxic mechanisms and key molecular targets of bisphenol A (BPA) in prostate cancer by integrating network toxicology, molecular docking, molecular dynamics simulations, and experimental validation, with a particular focus on elucidating the involvement of EMT and the PI3K/AKT signaling pathway in BPA-induced prostate cancer progression.

## 2 Materials and Methods

### 2.1 Initial Network-Based Toxicity Profiling of BPA

Network-based toxicity profiling of BPA was performed using multiple *in silico* toxicity prediction platforms, including ADMETlab 2.0 (Shanghai Institute of Materia Medica, Shanghai, China), ProTox-II (Charité–Universitätsmedizin Berlin, Berlin, Germany), and Vnn-ADMET (University of Milano-Bicocca, Milan, Italy). Each platform was used to predict BPA-associated toxicological properties and potential target genes based on its chemical structure. The predicted results were integrated and used for subsequent network and enrichment analyses.

### 2.2 Curation of a BPA Targets Library

The standardized SMILES and molecular structure of BPA were obtained from the PubChem database (National Center for Biotechnology Information, Bethesda, MD, USA; https://pubchem.ncbi.nlm.nih.gov; PubChem CID: 6623). Potential BPA targets were retrieved from the ChEMBL database (version 33; European Bioinformatics Institute, Hinxton, UK; https://www.ebi.ac.uk/chembl) and the STITCH database (version 5.0; Swiss Institute of Bioinformatics, Lausanne, Switzerland; http://stitch.embl.de) [27]. SwissTargetPrediction (Swiss Institute of Bioinformatics, Lausanne, Switzerland; http://www.swisstargetprediction.ch) was used for supplementary target identification. Targets obtained from these databases were collected based on reported or predicted compound–protein interactions. After merging the target lists and removing duplicate entries, a non-redundant BPA-related target library was generated for subsequent analyses.

### 2.3 Acquisition of Disease-Related Targets

Prostate cancer-related target genes were obtained from the GeneCards database (https://www.genecards.org) and the Online Mendelian Inheritance in Man (OMIM) database (https://omim.org). The intersection between prostate cancer–associated genes and BPA-related target genes was identified using the R programming environment. Only exact overlaps based on official gene symbols were considered. Genes present in both datasets were defined as potential targets involved in the toxic effects of BPA on prostate cancer development.

### 2.4 Construction of a Protein-Protein Interaction (PPI) Network

Protein-protein interaction (PPI) networks were constructed using the STRING database (version 11.5; Swiss Institute of Bioinformatics, Lausanne, Switzerland; https://string-db.org/) [28]. Common targets identified from the Venn diagram were uploaded to the STRING platform, with the organism restricted to *Homo sapiens*. To ensure high-confidence interactions and reduce false-positive connections, a minimum required interaction confidence score of 0.7 (high confidence) was applied. The resulting PPI interaction data were downloaded in TSV format and imported into cytoscape software (version 3.9.1; National Institute of General Medical Sciences, Bethesda, MD, USA) for network visualization and topological analysis using the Network Analyzer plugin. Topological parameters, including degree centrality, were calculated to identify key nodes within the network. Nodes with degree values greater than or equal to twice the median degree were defined as core hub targets, a commonly used criterion for identifying highly connected and potentially functionally important nodes in scale-free PPI networks [29,30].

### 2.5 GO and KEGG Pathway Analysis

Gene Ontology (GO) and Kyoto Encyclopedia of Genes and Genomes (KEGG) enrichment analyses of BPA-related target genes were performed using the clusterProfiler R package (version 4.0.5). GO enrichment analyses were conducted separately for the biological process (BP), cellular component (CC), and molecular function (MF) categories. The background gene set consisted of all annotated human protein-coding genes. GO and KEGG enrichment analyses were performed using the enrich GO and KEGG functions, respectively, with the organism parameter set to “hsa”. Multiple testing correction was applied using the Benjamini–Hochberg false discovery rate (FDR) method, and terms with an adjusted *p* value <0.05 were considered statistically significant. Enrichment results were visualized using the ggplot2 R package (version 3.3.6) in R software (version 3.5.2; R Foundation for Statistical Computing, Vienna, Austria).

### 2.6 Bioinformatics and Statistical Analysis of Core Target Genes

Gene expression data of AR, MMP2, MMP9, KLK3, and HIF1A in prostate cancer were obtained from The Cancer Genome Atlas (TCGA) database (https://www.cancer.gov/tcga). The transcriptomic data used in this analysis were RNA-sequencing (RNA-Seq) data, including 481 tumor samples and 51 normal samples, for a total of 532 samples analyzed. Differential expression analysis between tumor and normal tissues was performed using R software (version 3.5.2). The “limma” package was used for statistical analysis. The Benjamini–Hochberg method was applied for multiple testing correction to control the false discovery rate (FDR). Principal component analysis (PCA) was conducted using the “prcomp” function in R to evaluate sample distribution patterns based on the expression profiles of the five genes. The data were centered and scaled prior to analysis. PCA was used to assess sample distribution and expression pattern differences between tumor and normal tissues. Correlation analysis among these genes was performed using Pearson correlation coefficients based on normalized gene expression data, and the statistical significance of the correlations was evaluated using two-tailed *p* values, with *p* < 0.05 considered statistically significant. The results were visualized using the “corrplot” R package.

### 2.7 Immune Infiltration Analysis

The relative proportions of tumor-infiltrating immune cells were estimated using the CIBERSORT algorithm based on RNA-Seq transcriptomic data from TCGA prostate cancer cohort. A total of 185 prostate cancer tumor samples and 51 normal samples were included in the immune infiltration analysis. Gene expression matrices were uploaded to the CIBERSORT web portal using the LM22 immune cell signature matrix, and 1000 permutations were performed. Prior to input into CIBERSORT, gene expression data were normalized using the transcripts per million (TPM) method to ensure comparability across samples. No batch effect correction was applied, as all data were derived from the same cohort. Samples with CIBERSORT *p* < 0.05 were considered eligible for subsequent analyses, as this threshold is widely used to ensure statistically reliable immune cell deconvolution results. Differences in immune cell fractions between tumor and normal tissues were evaluated using the Wilcoxon rank-sum test and visualized using R software (version 3.5.2).

### 2.8 Survival and Prognostic Analysis

Survival analysis of core target genes was performed using transcriptomic and clinical data from The Cancer Genome Atlas (TCGA) prostate cancer cohort. The transcriptomic data used in this analysis were RNA-Seq transcriptomic data, and a total of 185 prostate cancer patients with available survival information were included. Patients were stratified into high- and low-expression groups according to the median expression level of each gene. Genes with insufficient expression levels across samples were excluded prior to stratification. Kaplan–Meier survival curves were generated to evaluate overall survival differences between groups, and statistical significance was assessed using the log-rank test. Patients lost to follow-up or without events were treated as censored. To evaluate the prognostic performance of the target genes, time-dependent receiver operating characteristic (ROC) curve analysis was performed using the survivalROC R package, and the area under the curve (AUC) values were calculated at the 3-year overall survival time point. All statistical analyses and visualizations were conducted using R software (version 3.5.2).

### 2.9 Molecular Docking

Three-dimensional structures of core target proteins (*Homo sapiens*) were obtained from the RCSB Protein Data Bank (https://www.rcsb.org) and preprocessed using PyMOL software (version 2.5.0; Schrödinger, LLC, New York, NY, USA) to remove crystallographic water molecules and co-crystallized ligands. Protein preparation was performed using AutoDockTools (ADT, version 1.5.7; The Scripps Research Institute, La Jolla, CA, USA). During protein preparation, polar hydrogen atoms were added, Gasteiger partial charges were assigned, and non-polar hydrogens were merged. Missing residues were not modeled, and only resolved regions in the crystal structures were used for docking. The three-dimensional structure of bisphenol A (BPA) was retrieved from the PubChem database (https://pubchem.ncbi.nlm.nih.gov) and subjected to energy minimization prior to docking. Molecular docking was performed using AutoDock Vina (version 1.2.3; The Scripps Research Institute). Docking grid boxes were defined to fully cover the active or predicted binding sites of the target proteins. For each protein–ligand complex, multiple docking poses were generated, and the binding affinity was evaluated based on the Vina scoring function (kcal/mol). The docking pose with the lowest binding free energy and a reasonable binding conformation was selected as the representative structure for further analysis. Discovery Studio Visualizer (version 2021; BIOVIA, Dassault Systèmes, San Diego, CA, USA) and PyMOL were used to visualize protein–ligand interactions and analyze binding modes.

### 2.10 Molecular Dynamics Studies

Molecular dynamics (MD) simulations of BPA complexes with AR, MMP2, MMP9, KLK3, and HIF1A were performed using GROMACS software (version 2022.4; University of Groningen, Groningen, The Netherlands). The Amber14SB force field was applied to proteins, and GAFF2 parameters were used for BPA ligands. Atomic partial charges for BPA were assigned using the AM1-BCC charge model as implemented in the Antechamber module of AmberTools. Systems were solvated in a TIP4P water box with a 1.2 nm buffer and neutralized with sodium and chloride ions. Long-range electrostatic interactions were treated using the Particle Mesh Ewald method. Energy minimization was conducted using the steepest descent algorithm, followed by equilibration under constant volume and constant pressure at 310 K. Production MD simulations were carried out for 100 ns with a 2 fs time step. Trajectories were analyzed for root mean square deviation (RMSD), root mean square fluctuation (RMSF), radius of gyration (Rg), solvent-accessible surface area (SASA), hydrogen bond formation, and free energy landscape (FEL). Binding free energies were estimated using the Molecular Mechanics Poisson-Boltzmann Surface Area (MM-PBSA) method.

### 2.11 Cell Culture and Treatment

PC3 and DU145 human prostate cancer cell lines were obtained from Shanghai Model Organisms Company (Shanghai, China; catalog no. NM-H003 and NM-H004). Cell line authentication was performed by short tandem repeat (STR) profiling, and both cell lines were confirmed to be free of mycoplasma contamination by routine PCR-based testing prior to use. Cells were cultured in RPMI-1640 medium (Gibco, Grand Island, NY, USA; catalog no. 11875-093) supplemented with 10% fetal bovine serum (FBS; Gibco, Grand Island, NY, USA; catalog no. 10099-141). Cells were maintained at 37°C in a humidified incubator with 5% CO_2_, Vehicle solutions were prepared using dimethyl sulfoxide (DMSO; Sigma-Aldrich, St. Louis, MO, USA; catalog no. D2650), and the final concentration of DMSO was kept consistent across all treatment groups (0.1% v/v).

### 2.12 Invasion and Migration Assays

Cell invasion and migration assays were performed using Transwell chambers (24-well format, 8-μm pore size; Corning Costar, Corning, NY, USA; Cat. No. 3422). For invasion assays, the upper surface of the inserts was coated with Matrigel Basement Membrane Matrix (BD Biosciences, San Jose, CA, USA; Cat. No. 354234) diluted 1:8 in serum-free RPMI-1640 medium (Gibco, Grand Island, NY, USA; catalog no. 11875-093) and incubated at 37°C for 12 h. Migration assays were conducted using uncoated inserts. Bisphenol A (BPA; Sigma-Aldrich, St. Louis, MO, USA; Cat. No. 239658) was dissolved in DMSO to prepare stock solutions. PC-3 and DU145 cells were pretreated with BPA at final concentrations of 10, 100, or 1000 nM for 24 h. For inhibitor experiments, cells were treated with the PI3K inhibitor LY294002 (20 μM) alone or in combination with BPA (100 nM) for 24 h prior to transwell assays. After treatment, cells were harvested, resuspended in serum-free medium, and seeded into the upper chambers at a density of 5 × 10^4^ cells per well in 200 μL. The lower chambers were filled with 600 μL of complete medium containing 10% fetal bovine serum (FBS; Gibco, Thermo Fisher Scientific, Waltham, MA, USA; Cat. No. 10099-141) as a chemoattractant. After incubation for 24 h at 37°C in a humidified incubator with 5% CO_2_, non-migrated or non-invaded cells on the upper surface of the membrane were gently removed using cotton swabs. Cells that had migrated or invaded to the lower surface were fixed with 4% paraformaldehyde for 15 min, stained with 0.1% crystal violet solution (Sigma-Aldrich; Cat. No. C0775) for 20 min, and washed with distilled water. Images were captured under an inverted microscope, and cells were counted in five randomly selected fields per insert. All experiments were performed in triplicate.

### 2.13 Western Blotting

Western blotting was performed according to a standard protocol with minor modifications. Cells or tumor tissues were lysed in RIPA buffer (Beyotime, Shanghai, China; catalog no. P0013B) supplemented with protease and phosphatase inhibitor cocktails (Roche, Switzerland). Protein concentrations were determined using a BCA protein assay kit (Thermo Fisher Scientific, USA). Equal amounts of protein (30 μg per lane) were separated by SDS–PAGE using 8%–12% polyacrylamide gels, depending on the molecular weight of the target proteins, and subsequently transferred onto PVDF membranes (0.45 μm; Millipore, USA). Membranes were blocked with 5% non-fat dry milk or 5% bovine serum albumin (BSA) in Tris-buffered saline containing 0.1% Tween-20 (TBST) for 1 h at room temperature and then incubated overnight at 4°C with primary antibodies diluted in blocking buffer. The following primary antibodies were used: GAPDH (CST #92310, 1:1000), Akt (CST #4691, 1:1000), phospho-Akt (Ser473; CST #4060, 1:1000), PI3K (CST #4292, 1:1000), phospho-PI3K (Tyr458; CST #17366, 1:1000), GSK-3β (CST #9315, 1:1000), phospho-GSK-3β (Ser9; CST #5558, 1:1000), MMP2 (CST #40994, 1:1000), MMP9 (CST #13667, 1:1000), N-cadherin (CST #13116, 1:1000), β-catenin (CST #8480, 1:1000), vimentin (CST #5471, 1:1000), Snail (CST #3879, 1:1000), Slug (CST #9585, 1:1000), and Twist1 (CST #90445, 1:1000). All primary antibodies were purchased from Cell Signaling Technology (Danvers, MA, USA).

After washing with TBST, membranes were incubated with HRP-conjugated secondary antibodies, including anti-rabbit IgG (Abcam ab288151, 1:5000) or anti-mouse IgG (Abcam ab205719, 1:5000), for 1 h at room temperature. Protein bands were visualized using an enhanced chemiluminescence (ECL) detection system (Thermo Fisher Scientific, USA) and captured with a ChemiDoc imaging system (Bio-Rad, USA). GAPDH was used as the internal loading control. Band intensities were quantified by densitometric analysis using ImageJ software (National Institutes of Health, USA), and the relative protein expression levels were normalized to GAPDH.

### 2.14 Animal Experiments

All animal experiments were conducted in accordance with the ARRIVE Essential 10 guidelines and were approved by the Animal Ethics Committee of Guizhou Provincial People’s Hospital (Approval No. 2021-069). Five-week-old male BALB/c nude mice (specific pathogen-free, SPF grade; body weight 18–22 g) were purchased from Hunan Silek Jingda Laboratory Animal Co., Ltd. (Hunan, China). Mice were housed under standard SPF conditions with controlled temperature (22°C ± 2°C), humidity (50%–60%), and a 12-h light/dark cycle, with free access to food and water. PC3 cells were harvested during the logarithmic growth phase, and a total of 1 × 10^6^ cells suspended in sterile phosphate-buffered saline (PBS) were intraperitoneally injected into each mouse to establish an intraperitoneal prostate cancer tumor dissemination model. Twenty-four mice were randomly assigned into four groups (n = 6 per group): control, BPA, LY294002, and BPA + LY294002. Bisphenol A (BPA; Sigma-Aldrich, St. Louis, MO, USA; catalog no. 239658) was dissolved in olive oil and administered by oral gavage once daily at a dose of 50 μg/kg body weight. The PI3K inhibitor LY294002 (Selleck Chemicals, Houston, TX, USA; catalog no. S1105) was dissolved in dimethyl sulfoxide (DMSO) and further diluted with sterile saline, and administered intraperitoneally once daily at a dose of 50 mg/kg [31]. For the combination treatment group, mice received BPA (50 μg/kg/day, oral gavage) together with LY294002 (50 mg/kg/day, intraperitoneal injection) simultaneously throughout the treatment period. Control mice received equivalent volumes of the corresponding vehicle solutions. The volumes used for oral gavage and intraperitoneal injection were within standard and acceptable limits for mice. After 5 weeks of treatment, mice were euthanized by carbon dioxide inhalation followed by cervical dislocation to confirm death. Tumor lesions within the abdominal cavity were carefully collected, photographed, counted, and weighed. The total tumor burden per mouse was recorded for subsequent analysis.

### 2.15 Statistical Methods

All treatments were performed independently at least three times, with the results being presented as means ± standard deviation (SD). Statistical analyses were performed using R software (version 3.5.2; R Foundation for Statistical Computing, Vienna, Austria), SPSS software (version 19.0; IBM Corp, Armonk, NY, USA), and GraphPad Prism (version 8.0; GraphPad Software, San Diego, CA, USA). Comparisons between two independent groups were made using Student’s *t*-test or the Mann-Whitney U test. A two-tailed *p* value < 0.05 was considered statistically significant.

## 3 Results

### 3.1 Identification of Targets Related to BPA-Induced Prostate Toxicity

**Figure 1:**
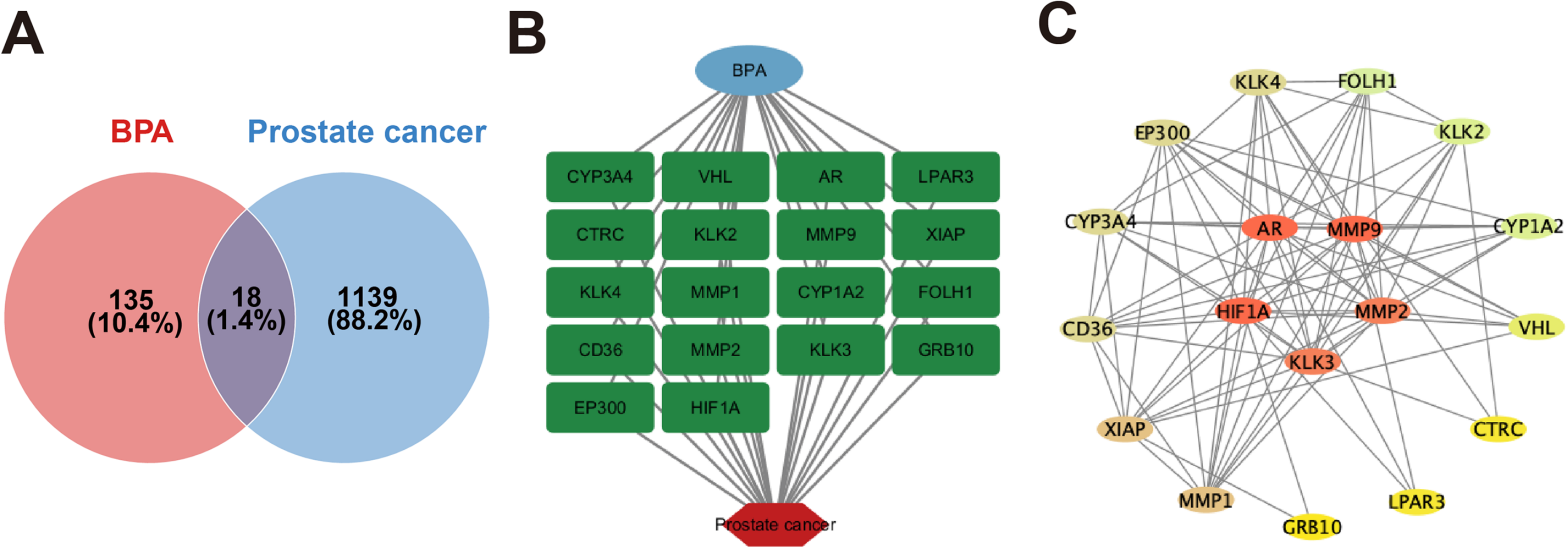
Venn diagram of potential Bisphenol A (BPA)-induced prostate cancer targets. (**A**,**B**) Associated target genes of BPA-induced prostate cancer. (**C**) Protein-protein interaction network of BPA-induced prostate cancer.

By integrating data from the ChEMBL and STITCH databases, SwissTargetPrediction tools, and 1157 prostate cancer-related genes retrieved from the GeneCards and OMIM databases, a total of 153 BPA-related targets were identified. Venn diagram analysis revealed 18 overlapping targets between BPA-related targets and prostate cancer-associated genes (Fig. 1A), and the list of intersecting genes is shown in Fig. 1B. These targets were further mapped to a PPI network using the STRING database. Network topology analysis performed with Cytoscape identified AR, MMP9, MMP2, KLK3, and HIF1A as nodes with the highest degree values in the PPI network (Fig. 1C).

### 3.2 Analysis of Target Gene Enrichment

**Figure 2:**
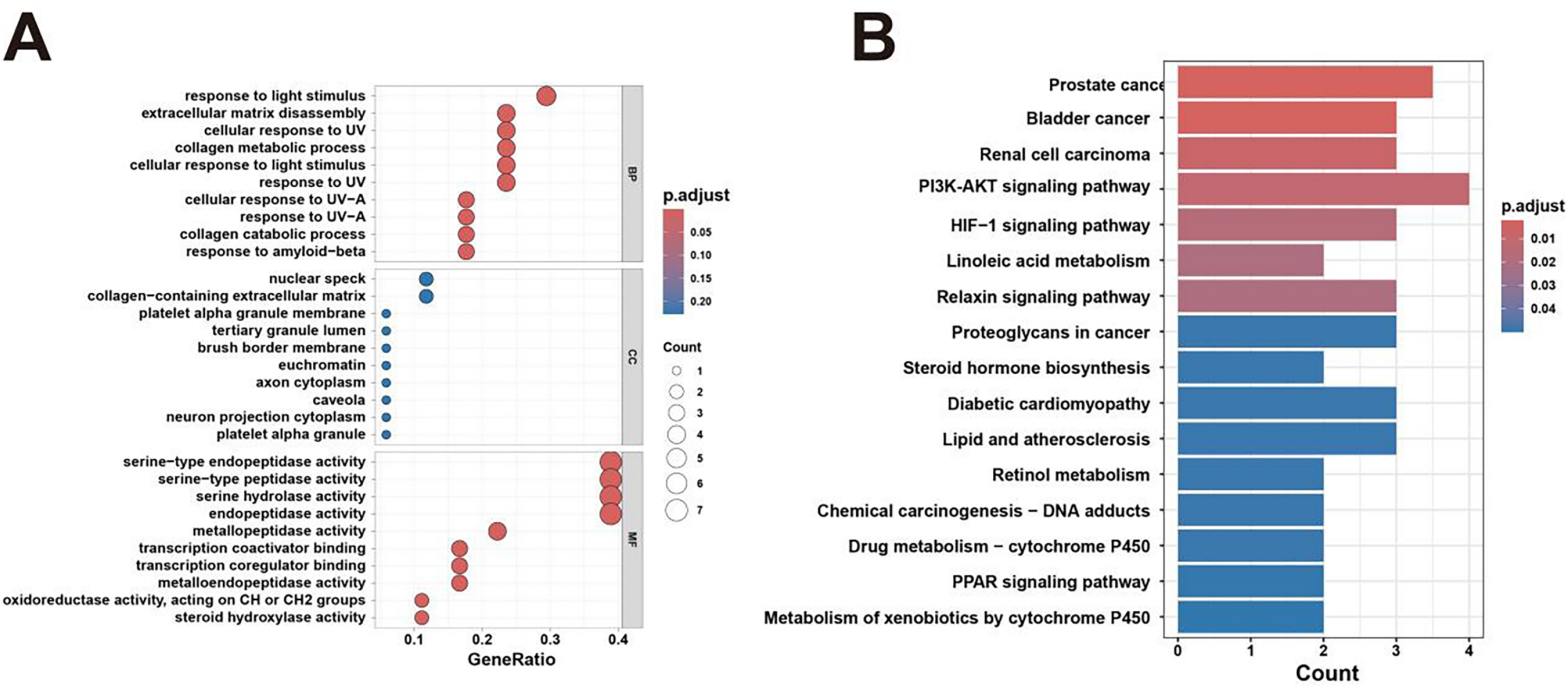
Functional enrichment analysis of prostate cancer. (**A**) GO enrichment analysis showing significantly enriched biological processes, cellular components, and molecular functions. (**B**) KEGG pathway enrichment analysis showing significantly enriched signaling pathways.

GO enrichment analysis showed that the intersecting target genes were mainly enriched in biological processes related to extracellular matrix disassembly and response to light stimulus. Cellular component analysis indicated significant enrichment in extracellular matrix-associated terms, granule lumen, euchromatin, and border membrane, while molecular function analysis revealed enrichment in functions associated with protein binding and receptor activity (Fig. 2A). KEGG pathway analysis further demonstrated that these target genes were enriched in metabolism- and lipid metabolism-related pathways, proteoglycans, atherosclerosis, chemical carcinogenesis, and cancer-associated pathways, including the PI3K/AKT signaling pathway (Fig. 2B), suggesting a potential key role of this pathway in BPA-induced prostate cancer progression.

### 3.3 Molecular Docking of BPA with Core Target Genes

**Figure 3:**
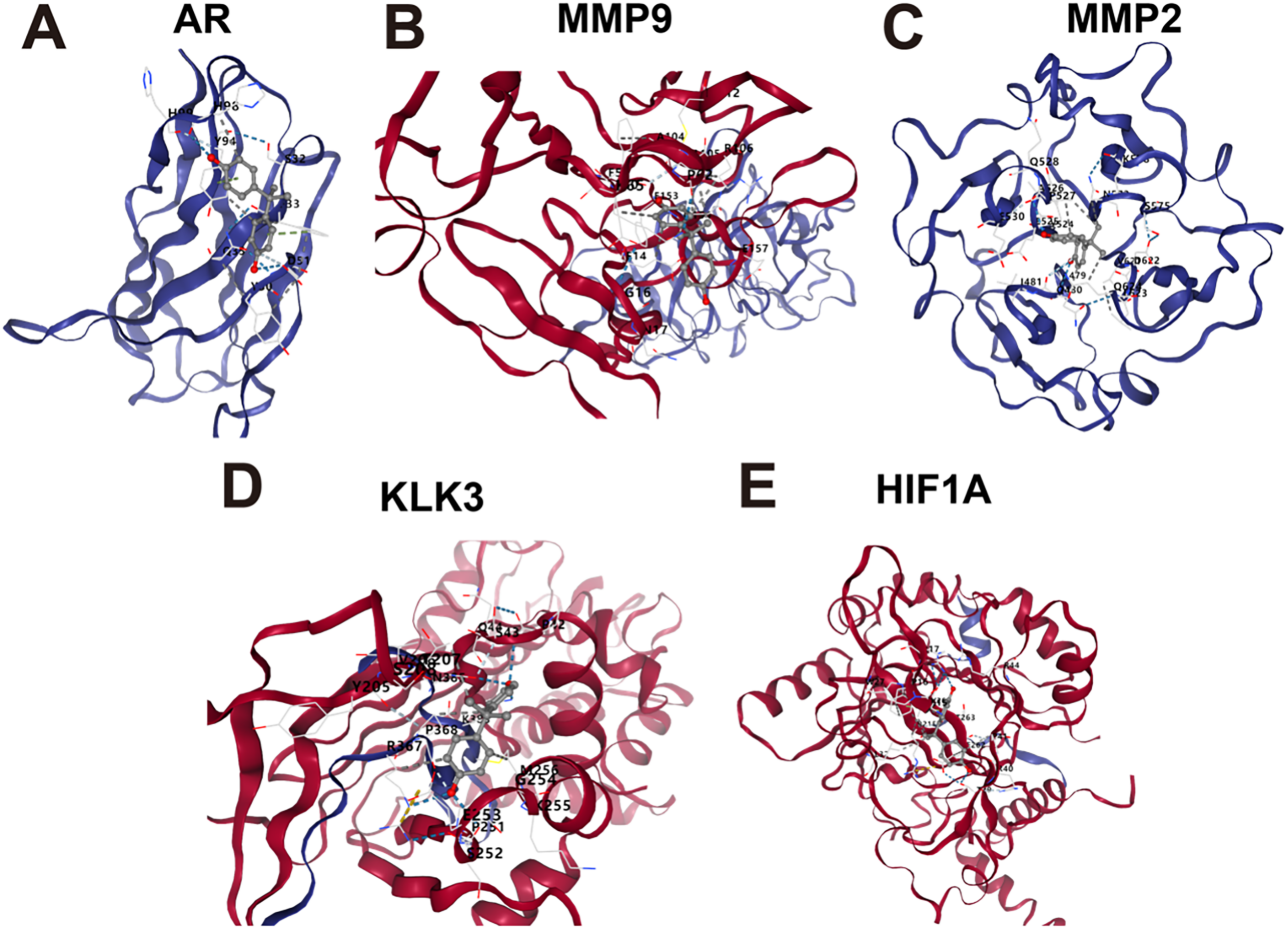
Molecular docking results showing the interactions between the five target proteins and BPA. (**A**) BPA and AR, (**B**) BPA and MMP9, (**C**) BPA and MMP2, (**D**) BPA and KLK3, (**E**) BPA and HIF1A.

Molecular docking analyses were performed to evaluate the interactions between BPA and five core target proteins (AR, MMP9, MMP2, KLK3, and HIF1A). Docking results showed that BPA exhibited low binding energies with all five proteins, indicating favorable binding conformations in the predicted complexes (Fig. 3A–E).

### 3.4 Further Analysis of Five Candidate Target Genes

**Figure 4:**
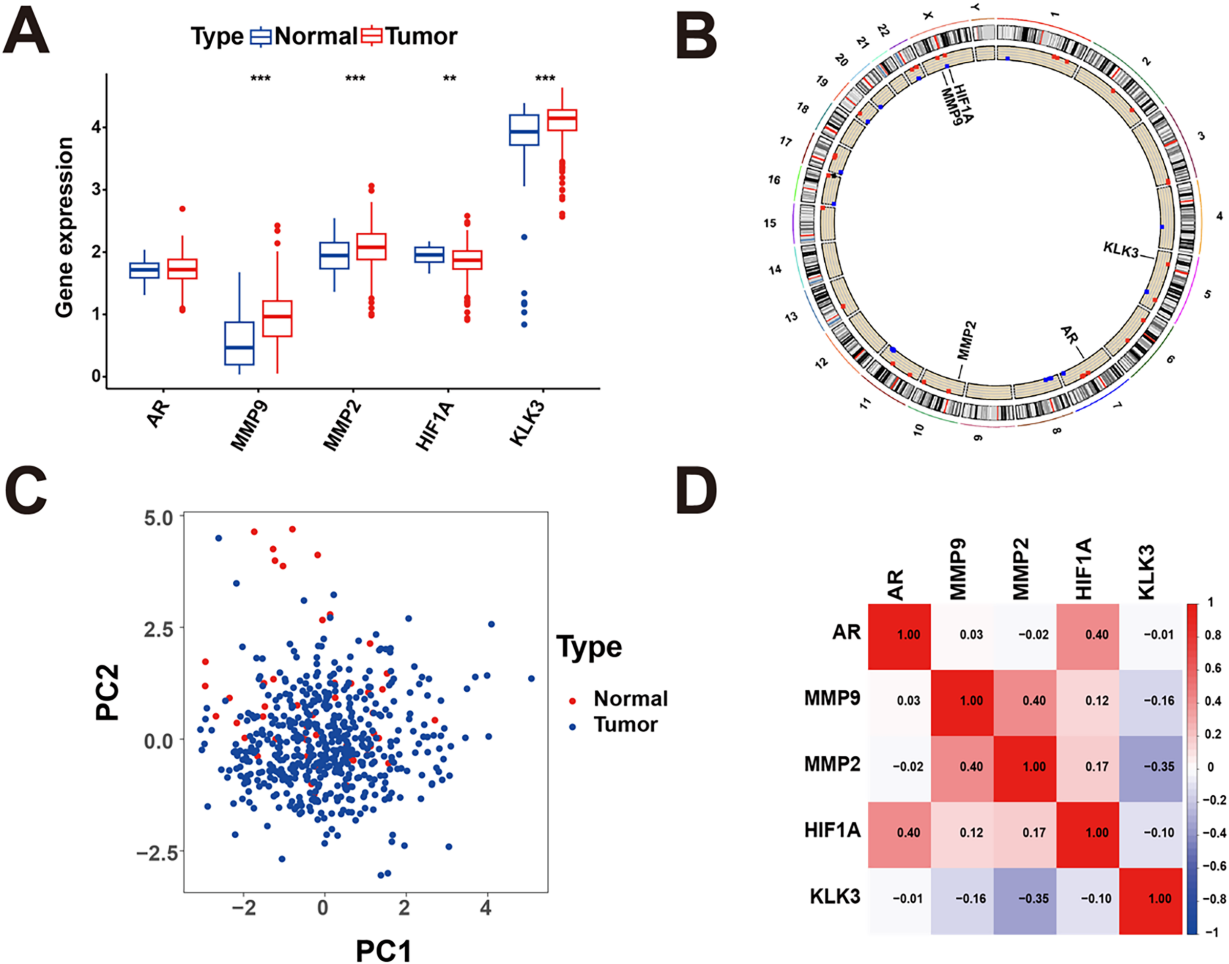
Relative expression of AR, MMP2, MMP9, KLK3, and HIF1A. (**A**) The expression levels of AR, MMP2, MMP9, KLK3, and HIF1A in normal and tumor samples. (**B**) Chromosomal locations of AR, MMP9, MMP2, KLK3, and HIF1A. (**C**) Principal component analysis showing a clear distinction between normal and tumor samples based on the five identified proteins. (**D**) Correlations among AR, MMP9, MMP2, KLK3, and HIF1A. **p < 0.01, ***p < 0.001.

Among the assessed target proteins, the expression of AR, MMP2, MMP9, and KLK3 was higher in prostate tumor tissues compared with normal tissues, whereas HIF1A showed lower expression in tumor samples (Fig. 4A). The chromosomal locations of the five genes are shown in Fig. 4B. Principal component analysis based on the expression of these genes clearly separated tumor samples from normal samples (Fig. 4C). Correlation analysis further revealed strong correlations among the five genes in prostate cancer samples (Fig. 4D).

### 3.5 Distribution of Immune Cells in Prostate Cancer

**Figure A1:**
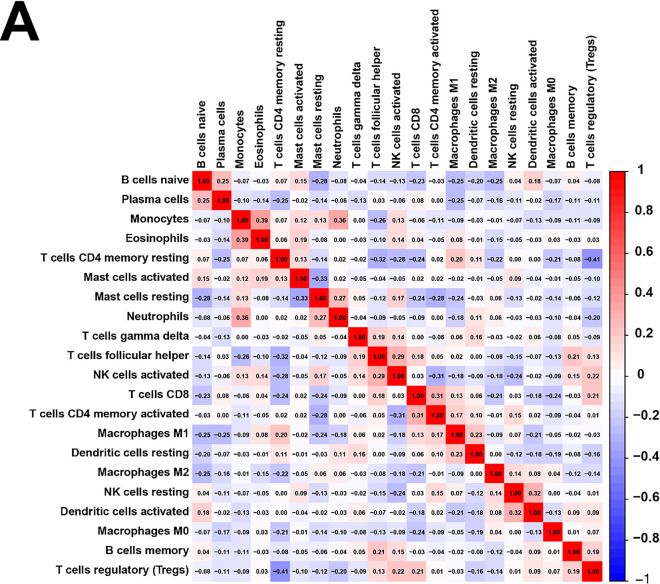

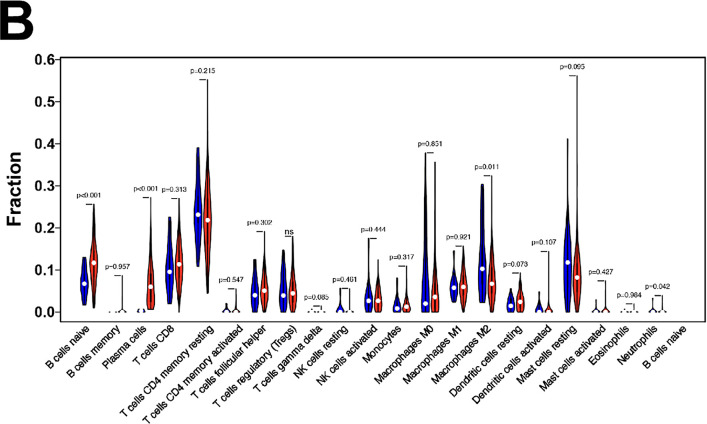
Immune cell distribution in prostate cancer. (**A**) Characteristics of immune cell infiltration. (**B**) Immune cell infiltration differences were observed between the normal and tumor groups. ns: not significant.

**Figure A2:**
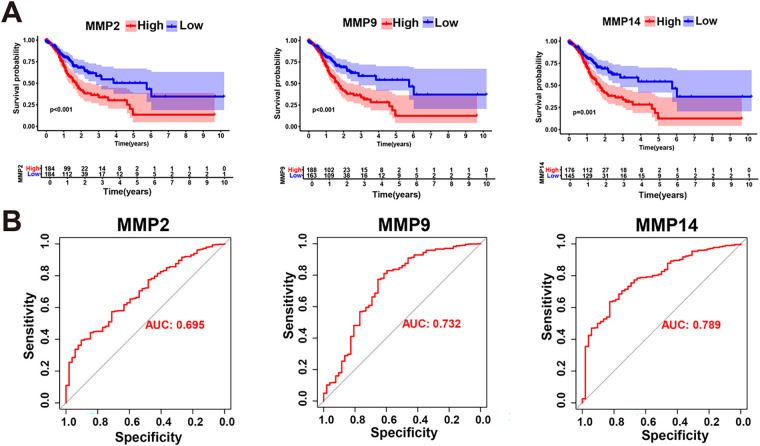
Survival analysis based on prognostic model grouping. (**A**) Kaplan-Meier survival analysis of high- and low-expression groups. (**B**) Time-dependent ROC curves. ROC: receiver operating characteristic.

To examine the association between immunity and prostate cancer, we determined the relative proportions of 22 immune cell types in different samples. Comparative analysis between tumor and normal tissues showed increased infiltration of M0 macrophages, naïve B cells, and CD8^+^ T cells in tumor samples, whereas M2 macrophages exhibited lower infiltration levels (Appendix A Fig. A1A,B). No significant differences were observed for other immune cell types. Kaplan-Meier survival analysis indicated that higher expression levels of MMP2, MMP9, and MMP14 were associated with poorer overall survival in patients with prostate cancer (Appendix A Fig. A2A). Moreover, receiver operating characteristic (ROC) curve analysis yielded area under the curve (AUC) values of 0.695 for MMP2, 0.732 for MMP9, and 0.789 for MMP14, indicating moderate prognostic performance in prostate cancer patients (Appendix A Fig. A2B). Survival and ROC analyses were based on TCGA prostate cancer cohort data.

### 3.6 Molecular Dynamics Simulation

**Figure A3:**
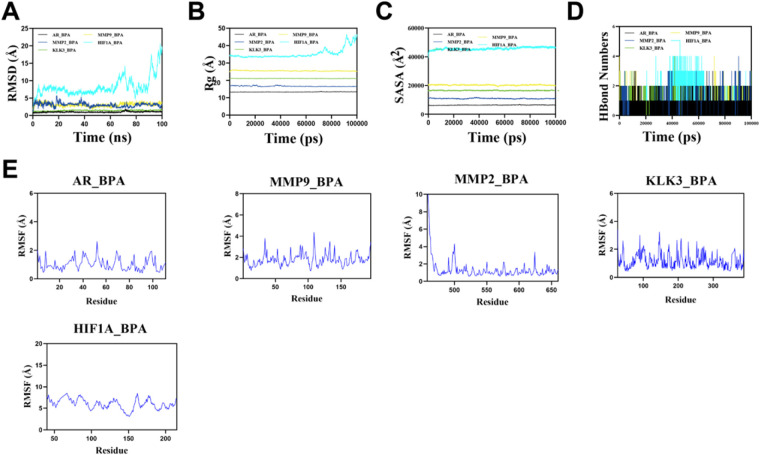
Molecular dynamics simulation. (**A**) represents the RMSD curve; (**B**) represents the Rg curve; (**C**) represents the SASA curve; (**D**) represents fluctuations in the number of hydrogen bonds; and (**E**) represents the RMSF curve.

To assess the stability of BPA-protein interactions, MD simulations were performed for BPA complexed with AR, MMP9, MMP2, KLK3, and HIF1A. RMSD analysis showed that the AR_BPA, MMP2_BPA, KLK3_BPA, and MMP9_BPA complexes reached equilibrium after approximately 20 ns and fluctuated below 3 Å, whereas the HIF1A_BPA complex exhibited delayed convergence, reaching a relatively stable regime between 30 and 60 ns with larger RMSD fluctuations below 18 Å (Appendix A Fig. A3A). The radius of gyration (Rg) and solvent-accessible surface area (SASA) analyses indicated relatively stable conformations for all complexes except HIF1A_BPA (Appendix A Fig. A3B,C). Hydrogen bond analysis showed that 0–5 hydrogen bonds were formed between BPA and the target proteins during the simulations (Appendix A Fig. A3D). RMSF analysis revealed that most residues fluctuated within 0.2–4 Å (Appendix A Fig. A3E).

**Figure 5:**
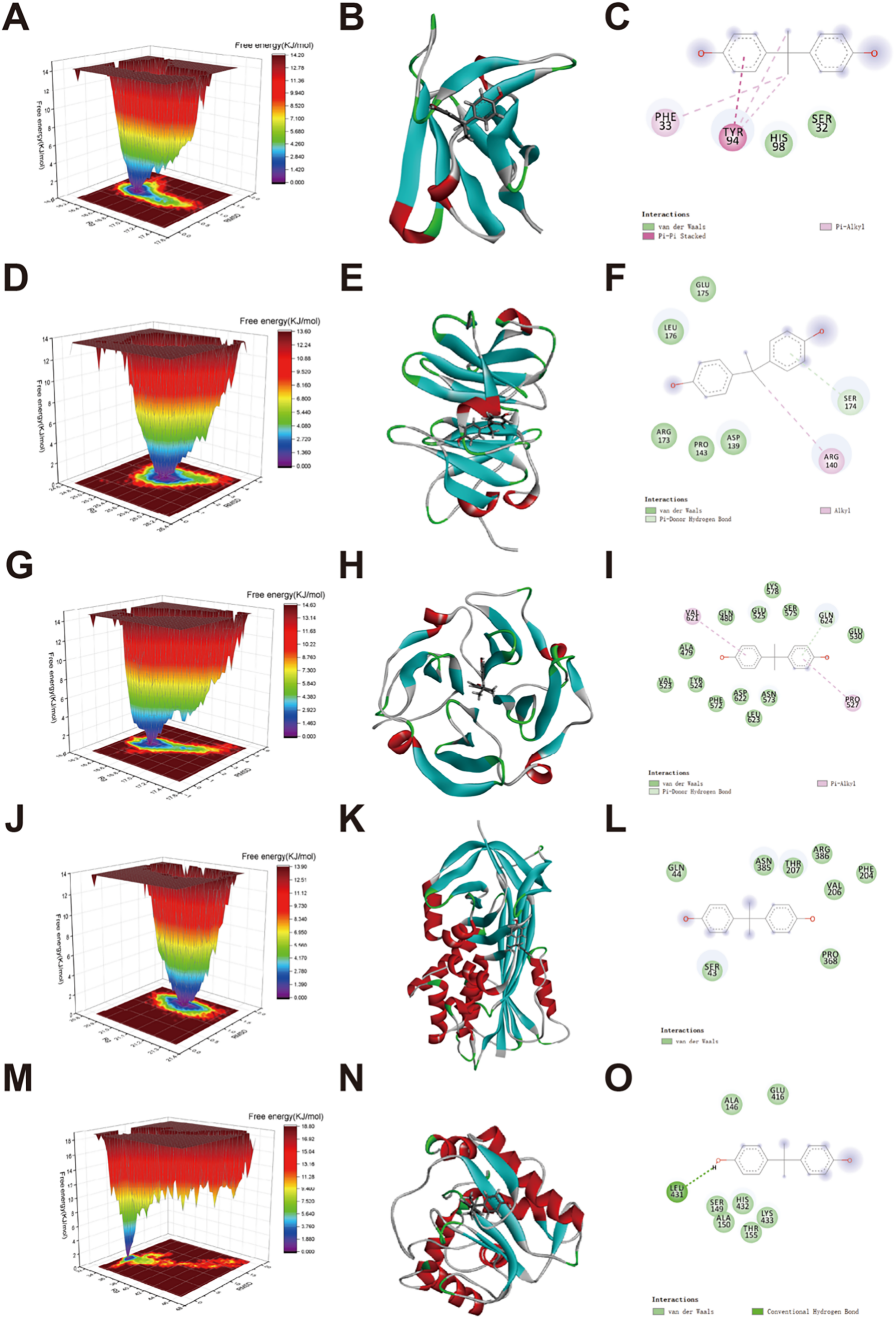
Free-energy landscape analysis of BPA-target protein complexes. (**A**,**D**,**G**,**J**,**M**) represent a free-energy surface; (**B**,**E**,**H**,**K**,**N**) represent minimal energy conformations; (**C**,**F**,**I**,**L**,**O**) represent protein-ligand interaction profiles. (**A**–**C**) BPA-AR, (**D**–**F**) BPA-MMP9, (**G**–**I**) BPA-MMP2, (**J**–**L**) BPA-KLK3, and (**M**–**O**) BPA-HIF1A.

**Figure 6:**
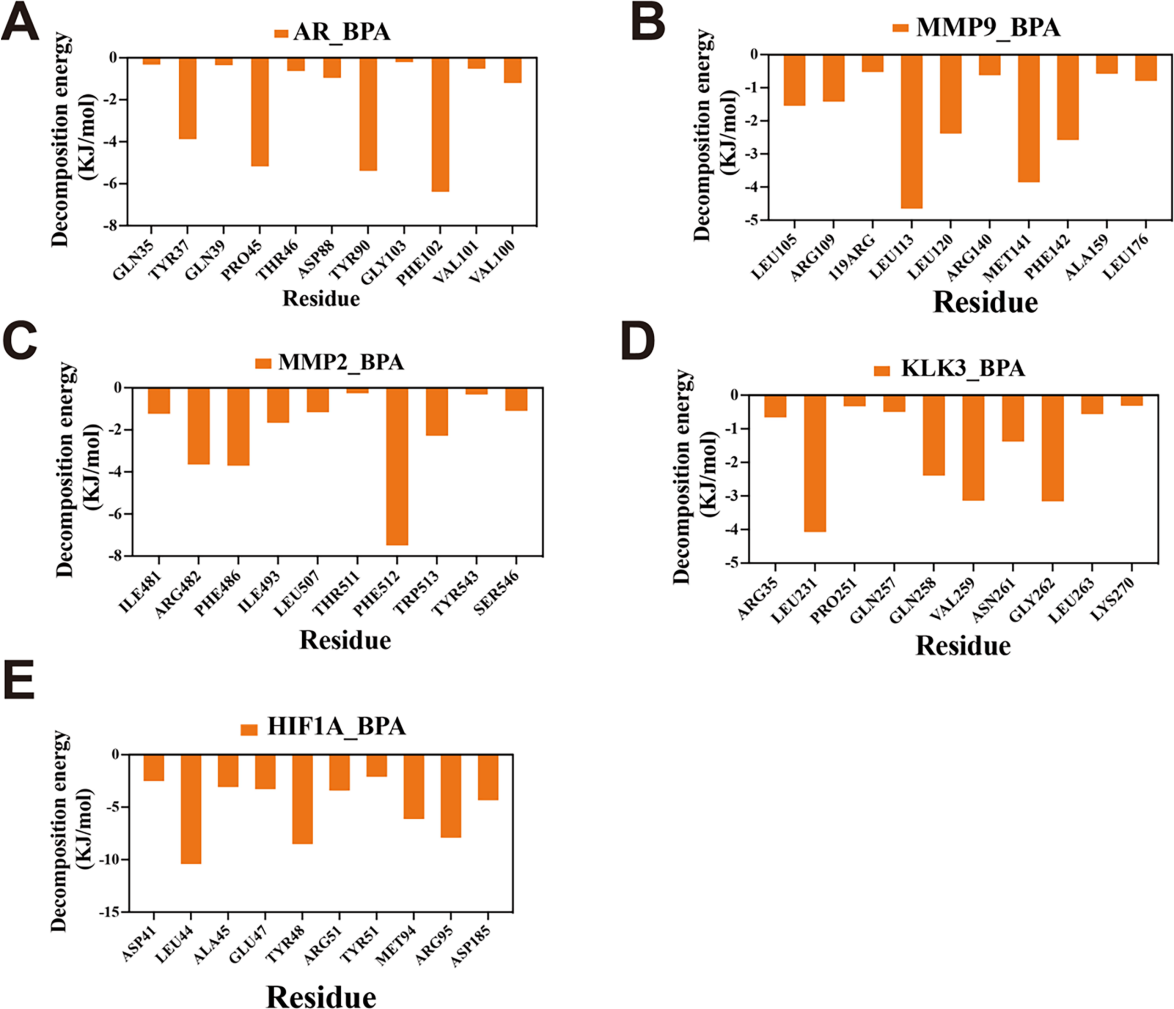
Decomposition analysis of the free energy of BPA-target protein complexes. (**A**) BPA-AR, (**B**) BPA-MMP9, (**C**) BPA-MMP2, (**D**) BPA-KLK3, and (**E**) BPA-HIF1A.

Free energy landscape (FEL) analysis showed that the minimum energy states of the protein-ligand complexes occurred at approximately 20 ns. Specific residue-level interactions between BPA and each target protein were identified, as illustrated in Fig. 5A–O. Binding free energy calculations using the MM/PBSA method yielded values of −72.023 kJ/mol (AR_BPA), −20.641 kJ/mol (MMP9_BPA), −63.650 kJ/mol (MMP2_BPA), −56.097 kJ/mol (KLK3_BPA), and −115.506 kJ/mol (HIF1A_BPA) (Fig. 6A–E).

### 3.7 Effects of BPA Exposure on Prostate Cancer Cell Invasion and Migration

**Figure A4:**
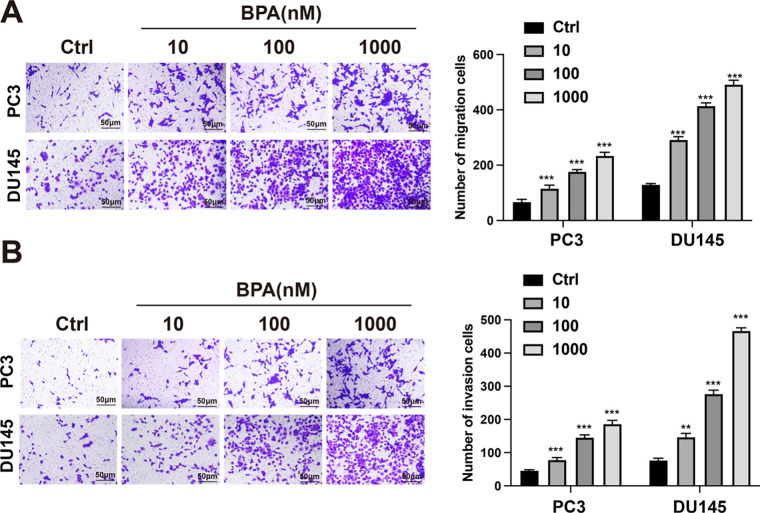
BPA promotes the migration and invasion of PC3 and DU145 cells. (**A**) PC3 and DU145 cells were seeded in the upper chamber of Transwell devices and treated with BPA for 24 h, followed by crystal violet staining of migrated cells. (**B**) Cells were seeded in the upper chamber of Transwell devices coated with Matrigel and allowed to invade for 24 h, after which, the cells were stained and counted. Results are expressed as the means ± SD of three replicate experiments. **p < 0.01, ***p < 0.001.

Cell invasion and migration assays were performed to assess the effects of BPA exposure on prostate cancer cells. BPA treatment for 24 h promoted the migration of PC3 and DU145 cells (Appendix A Fig. A4A) and significantly enhanced the invasive capacity of both cell lines (Appendix A Fig. A4B).

### 3.8 BPA Induces the Expression of Epithelial-Mesenchymal Transition (EMT) in Prostate Cancer

**Figure 7:**
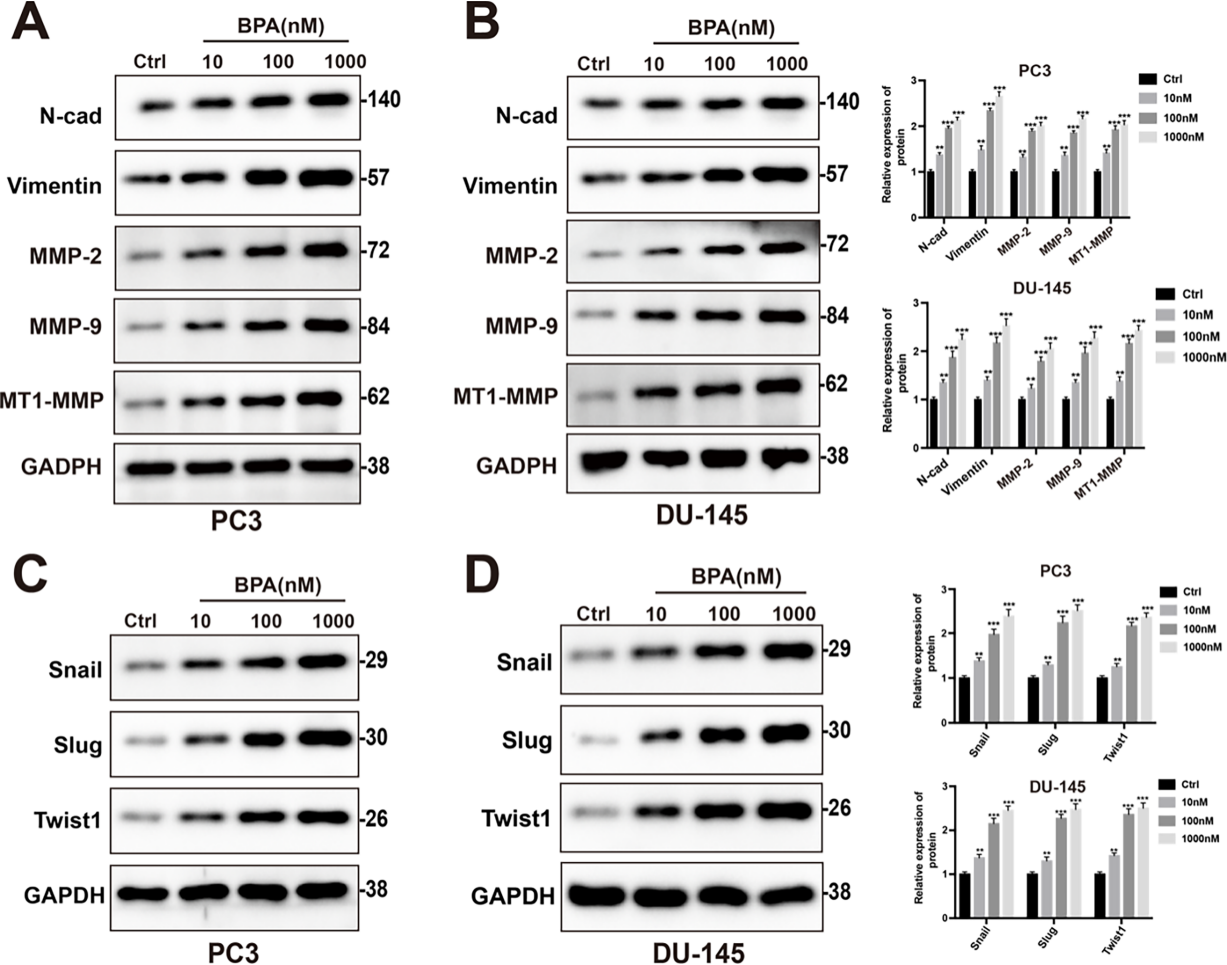
BPA regulates protein levels of EMT biomarkers and transcription factors in prostate cancer cells. (**A**,**B**) After 24 h of BPA treatment, the expression levels of N-cadherin, vimentin, MMP2, MMP9, and MT1-MMP were measured in PC3 and DU145 cells. (**C**,**D**) After 24 h of BPA treatment, the expression levels of Snail, Slug, and Twist1 were measured in PC3 and DU145 cells. Data are shown as the mean ± standard deviation of three independent experiments. **p < 0.01, ***p < 0.001.

Given the critical role of EMT in cancer metastasis, we examined the effects of BPA on EMT-related markers in prostate cancer cells. Western blot analysis showed that BPA treatment markedly increased the expression of N-cadherin, vimentin, MMP2, and MMP9, as well as the EMT-associated transcription factors Snail, Slug, and Twist1, in both PC3 and DU145 cells (Fig. 7A–D). In addition, BPA exposure significantly upregulated MT1-MMP (MMP14), a key regulator of pericellular matrix remodeling and tumor invasion [32]. Collectively, these results indicate that BPA promotes EMT-associated invasive phenotypes in prostate cancer cells *in vitro*.

### 3.9 BPA Regulates Prostate Cancer Cell EMT via the PI3K/AKT Pathway

**Figure A5:**
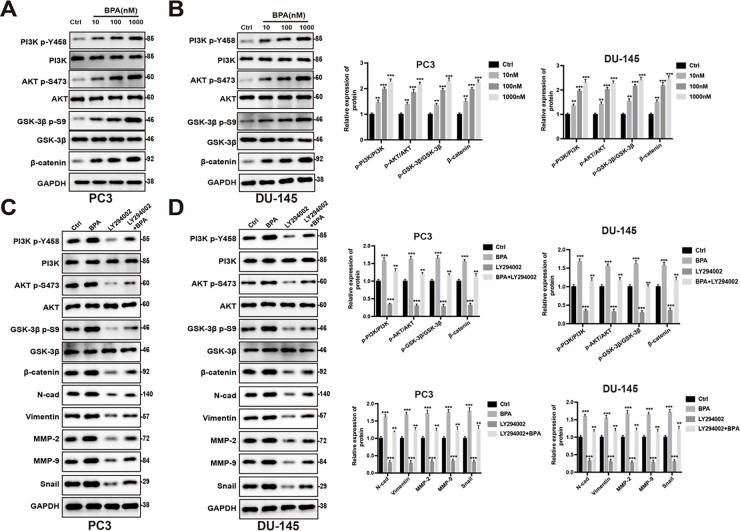
BPA activates the PI3K/AKT/GSK-3β/β-catenin signaling pathway.(**A**,**B**) BPA enhanced the PI3K/AKT/GSK-3β/β-catenin signaling pathway in a dose-dependent manner; cells were treated with BPA at different concentrations for 24 h. (**C**,**D**) BPA reversed the effect of the PI3K inhibitor LY294002 on the PI3K/AKT/GSK-3β/β-catenin signaling pathway in prostate cancer cells. Data are shown as the mean ± standard deviation of three independent experiments. **p < 0.01, ***p < 0.001.

**Figure A6:**
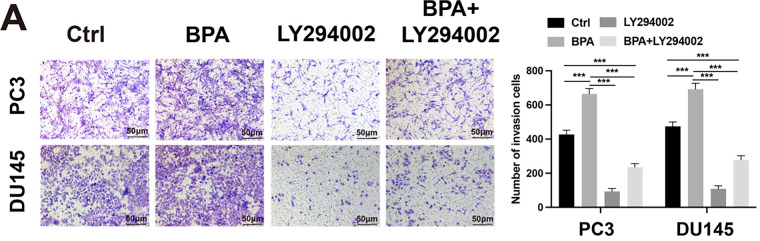

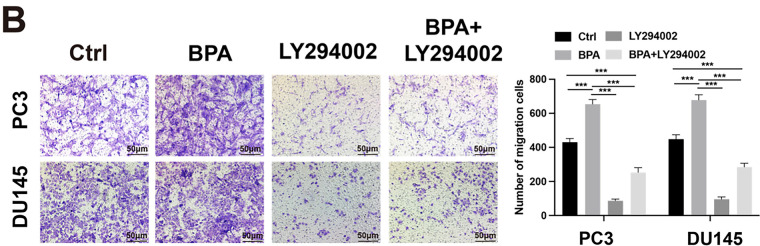
BPA promotes the migration and invasion of PC3 and DU145 cells. (**A**) PC3 and DU145 cells were seeded in the upper chamber of Transwell devices and treated with BPA for 24 h, followed by crystal violet staining of migrated cells. (**B**) Cells were seeded in the upper chamber of Transwell devices coated with Matrigel and allowed to invade for 24 h, after which, the cells were stained and counted. Results are expressed as the means ± SD of three replicate experiments. ***p < 0.001.

KEGG pathway enrichment analysis indicated that BPA-related targets were significantly associated with the PI3K/AKT signaling pathway, which is frequently dysregulated in prostate cancer. Consistently, western blot analysis showed that BPA treatment markedly increased the levels of phosphorylated PI3K, AKT, and GSK-3β, as well as β-catenin, in PC3 and DU145 cells (Appendix A Fig. A5A,B). To further clarify the involvement of PI3K/AKT signaling in BPA-induced EMT, cells were treated with the PI3K inhibitor LY294002(20 μM). LY294002 treatment reduced the phosphorylation of PI3K, AKT, and GSK-3β, accompanied by decreased expression of β-catenin, N-cadherin, vimentin, MMP2, MMP9, and Snail, whereas co-treatment with BPA markedly attenuated these inhibitory effects (Appendix A Fig. A5C,D). In line with these molecular changes, invasion and migration assays showed that BPA reversed the suppressive effects of LY294002 on prostate cancer cell motility (Appendix A Fig. A6A,B). Collectively, these results demonstrate that BPA promotes EMT and invasive potential in prostate cancer cells through activation of the PI3K/AKT signaling pathway.

### 3.10 BPA Promotes Prostate Cancer Cell Progression In Vivo

**Figure 8:**
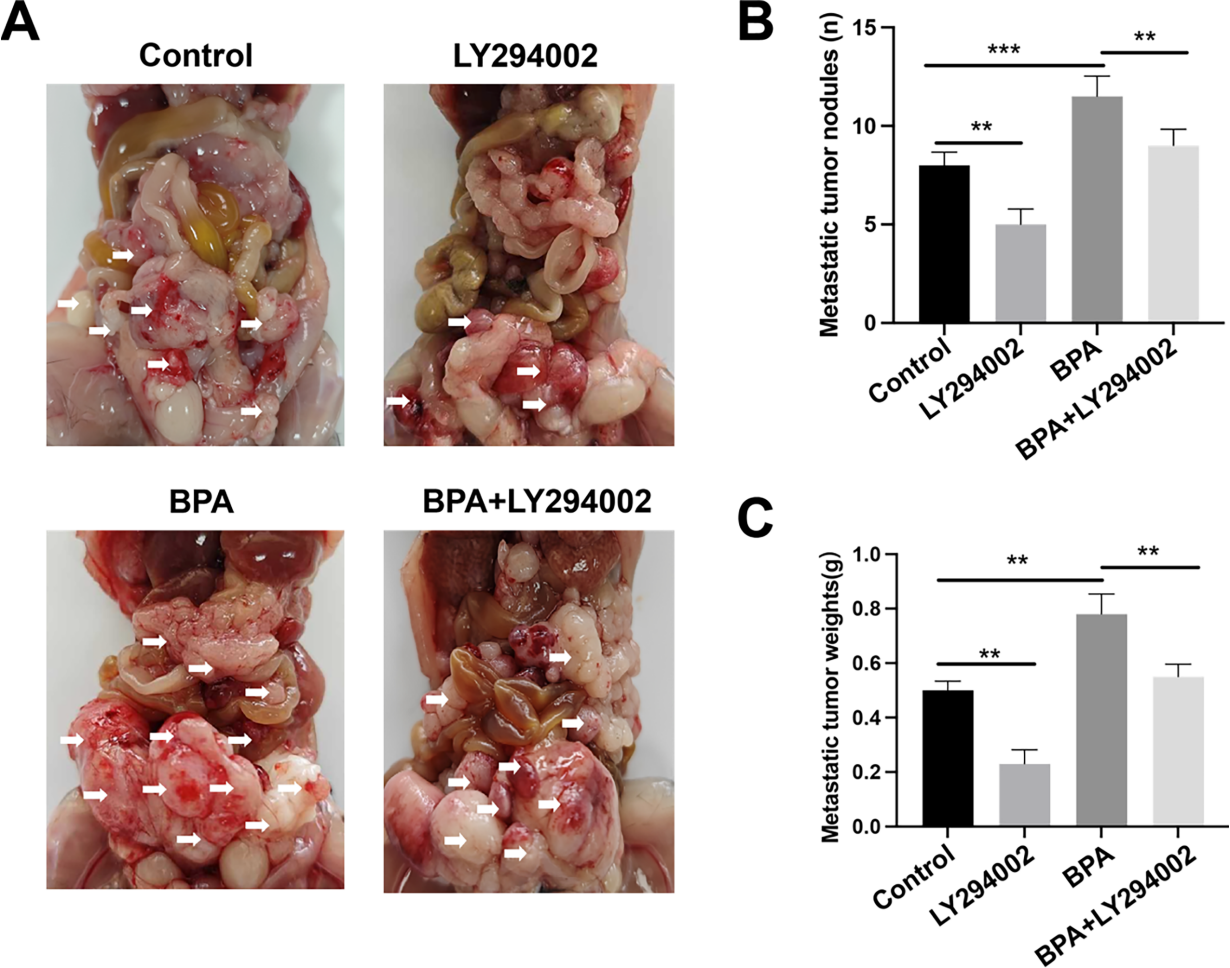
BPA promotes prostate cancer progression *in vivo*. (**A**) Representative images of tumor lesions within the abdominal cavity in different treatment groups, with arrows indicating tumor lesions. (**B**) Quantification of the number of tumor lesions per mouse. (**C**) Quantification of the total weight of tumor lesions per mouse. Data are shown as mean ± SD. **p < 0.01, ***p < 0.001.

To further evaluate the effects of BPA on prostate cancer progression *in vivo*, an intraperitoneal prostate cancer dissemination model was established in nude mice. After 5 weeks of treatment, multiple tumor lesions were observed within the abdominal cavity, including lesions associated with the intestines and peritoneum, indicating extensive tumor dissemination within the abdominal cavity *in vivo*. Compared with control mice, chronic BPA exposure markedly increased overall tumor burden, as reflected by both the number and total weight of tumor lesions per mouse, indicating that BPA promotes prostate cancer progression *in vivo*. Notably, treatment with the PI3K inhibitor LY294002 significantly reduced tumor burden and effectively suppressed BPA-induced prostate cancer progression (Fig. 8A–C).

## 4 Discussion

Prostate cancer is currently the most common cancer affecting men globally, with the highest incidence and second highest associated mortality among all male cancers [33,34]. Given the continuing lack of effective clinical treatments for advanced castration-resistant prostate cancer, in-depth research into its onset, development, and progression-related molecular mechanisms is essential to facilitate effective targeted treatment [35]. To this end, there has been notable growth in the number of studies investigating the associations between environmental contaminants and tumor progression. Despite regulatory restrictions on BPA use in many countries, lingering residues are still commonly detected owing to its environmental persistence and bioaccumulation in food chains [36]. Accumulating evidence has revealed a link between BPA exposure and prostate cancer, prompting further research into the underlying molecular mechanisms [37]. In the present study, we sought to gain further insight into the potential link between BPA exposure and prostate cancer using an investigative approach.

Based on existing literature and toxicity network analysis, we identified 18 potential targets associated with BPA-related prostate cancer using the ChEMBL, STITCH, GeneCards, and OMIM databases. Five core targets-AR, MMP9, MMP2, KLK3, and HIF1A-were subsequently identified using the STRING database and Cytoscape, all of which are involved in cell proliferation and tumor-associated biological processes. Functional and pathway enrichment analyses performed using the clusterProfiler R package highlighted the involvement of PI3K/AKT, HIF-1, and PPAR signaling pathways in prostate cancer, drug metabolism, lipid metabolism, atherosclerosis, and chemical carcinogenesis, supporting a role for BPA in prostate cancer progression.

Utilizing recent advances in bioinformatics platforms and computational approaches, network toxicology has emerged as an effective strategy for systematically exploring the molecular links between environmental toxicants and disease. In this study, we applied a network toxicology framework to identify BPA-related molecular signatures, highlighting AR, MMP2, MMP9, KLK3, and HIF1A as potential biomarkers. AR is a ligand-dependent transcription factor of the steroid nuclear receptor family that plays a critical role in prostate development and function [38]. During tumorigenesis, alterations in AR-dependent transcription are associated with the activation of pro-proliferative programs in prostate cancer cells [39]. In addition, AR contributes to metabolic reprogramming in prostate cancer by promoting glycolysis, mitochondrial respiration, and fatty acid β-oxidation, thereby supporting tumor growth [40,41]. Previous studies have investigated the association between MMP gene polymorphisms and the risk of prostate cancer [42,43], with MMPs reported to regulate key processes such as cell growth, inflammation, angiogenesis, and extracellular matrix (ECM) remodeling [44]. In particular, MMP2 and MMP9 have been associated with AR status and prostate cancer invasion [45]. By degrading ECM components and disrupting cell adhesion, MMPs facilitate tumor cell migration and metastasis [46,47]. Among MMP family members, MMP2 and MMP9 play pivotal roles in prostate cancer metastasis [48,49]. Consistent with these observations, our results demonstrated that BPA treatment significantly upregulated MMP2, MMP9, and MT1-MMP expression, accompanied by enhanced migratory and invasive capacities of prostate cancer cells, supporting a pro-invasive role of BPA.

KLK3, a prostate-specific antigen, is a well-established biomarker for prostate cancer and one of the most abundant proteins expressed in the prostate [50]. Experimental studies have demonstrated that KLK3 promotes prostate cancer cell and tumor growth and facilitates metastasis by stimulating tumor-associated angiogenesis and lymphangiogenesis through activation of VEGF-C and VEGF-D [51]. In parallel, HIF1A is a key regulator of cellular responses to hypoxia and tumor metabolic adaptation, particularly through its role in controlling glycolytic activity and cancer aggressiveness [52]. Notably, unlike AR, MMP2, MMP9, and KLK3, HIF1A exhibited reduced expression in prostate cancer tissues in our dataset, suggesting a distinct regulatory pattern. Given that HIF1A activity is predominantly regulated at post-transcriptional and protein stability levels, reduced mRNA expression does not necessarily reflect diminished functional activity. In line with this, molecular docking and molecular dynamics analyses supported a direct interaction between BPA and HIF1A, indicating that BPA may modulate HIF1A function in a transcription-independent manner. More broadly, docking analyses suggested that all five core targets are capable of forming stable interactions with BPA through hydrogen bonding and hydrophobic forces, supporting their potential involvement in BPA-associated molecular processes in prostate cancer.

Herein, we focused on the activities of EMT and the PI3K/AKT pathway in prostate cancer. It has been reported that cross-talk between MMPs and the PI3K/AKT pathway plays an important role in prostate cancer, leading to reduced survival. PI3K/AKT pathway signaling has also been identified as a key feature in different types of cancers, including breast cancer [53], prostate cancer [54], and tongue squamous cell carcinoma [55]. Our findings revealed that BPA promotes prostate cancer migration by activating the PI3K/AKT/GSK-3β signaling pathway, upregulating the expressions of N-cadherin, vimentin, MMP2, MMP9, and related transcription factors Snail, Slug, and Twist1. The promotive effect of BPA on prostate cancer cell migration was further verified using the PI3K inhibitor LY294002, indicating that the activation of the PI3K/AKT pathway may represent an important mechanism by which BPA enhances tumor progression. *In vivo* experiments showed that BPA exposure in an intraperitoneal tumor dissemination model promoted tumor progression, whereas treatment with the PI3K inhibitor LY294002 significantly reduced tumor burden. After establishing the central role of PI3K/AKT signaling, we further note that BPA may also influence carcinogenesis through additional mechanisms, including hormone receptor signaling, oxidative stress, hypoxia, and epigenetic regulation. Collectively, our findings provide valuable insights into the role of BPA in the progression of prostate cancer and serve as a foundation for the development of evaluation and preventive strategies for bisphenol-induced carcinogenesis.

Despite the strengths of this integrative study, several limitations should be acknowledged. Our analyses relied largely on publicly available databases, including CTD, GEO, and TCGA, and therefore did not directly assess BPA exposure sources or quantify BPA levels, which may limit causal inference. In addition, incomplete clinical annotation in some datasets, together with the relatively small and non-population-based nature of TCGA cohorts, may introduce selection bias and affect the generalizability of our findings. Although network toxicology, molecular docking, molecular dynamics simulations, and experimental assays collectively supported an association between BPA and prostate cancer progression, the identified core targets (AR, MMP2, MMP9, KLK3, and HIF1A) should be regarded as BPA-associated candidates rather than definitive causal mediators. Moreover, while docking and MD analyses suggested stable BPA-protein interactions and *in vivo* xenograft experiments indicated enhanced tumor progression and invasive potential, the precise molecular mechanisms underlying these observations were not fully delineated. Future studies incorporating genetic perturbation, quantitative exposure assessment, and *in vivo* molecular validation, as well as real-world datasets, will be essential to further clarify the mechanistic and translational relevance of BPA-associated signaling in prostate cancer.

## 5 Conclusion

In this study, we integrated network toxicology and molecular docking approaches to systematically investigate the potential effects of BPA on prostate cancer progression. A total of 153 candidate targets were initially identified, among which five core targets (AR, MMP2, MMP9, KLK3, and HIF1A) were highlighted as BPA-associated candidates. Molecular docking revealed negative binding energies, indicating favorable interactions between BPA and these proteins, which was further supported by molecular dynamics simulations showing stable binding. Moreover, *in vivo* experiments demonstrated that BPA exposure promotes prostate cancer progression through activation of PI3K/AKT-mediated EMT. Collectively, these findings elucidate potential molecular mechanisms underlying BPA-induced prostate cancer progression and underscore the utility of network toxicology and molecular modeling approaches in evaluating the biological effects of environmental pollutants.

## Data Availability

The data that support the findings of this study are available from the Corresponding Authors, [Xiaofei Fan, Jianguo Zhu], upon reasonable request.
